# Anterior Cutaneous Nerve Entrapment Syndrome From Glucagon-Like Peptide-1 Receptor Agonist Injection

**DOI:** 10.14309/crj.0000000000002168

**Published:** 2026-06-19

**Authors:** Neha Singh, Elisa Chaparro, Jordan Michael Shapiro, Emanuel Narcis Husu

**Affiliations:** 1 Department of Physical Medicine and Rehabilitation, Baylor College of Medicine, H. Ben Taub, Houston, TX; 2 Department of Physical Medicine and Rehabilitation, Larkin Community Hospital, South Miami, FL; 3 Gentle GI, PLLC, Houston, TX; 4 Department of Clinical Sciences, Chicago Medical School, Rosalind Franklin University of Medicine and Science, North Chicago, IL

**Keywords:** abdominal pain, glucagon-like peptide-1 receptor agonist, GLP-1 receptor agonist

## Abstract

Anterior cutaneous nerve entrapment syndrome (ACNES) is an underdiagnosed cause of abdominal pain. We report the first documented ACNES case following subcutaneous administration of glucagon-like peptide-1 receptor agonist. A 44-year-old man developed focal, neuropathic abdominal pain coinciding with initiation of tirzepatide injections. Clinical examination confirmed ACNES diagnosis, and pain improved after relocating injections away from the abdomen and initiating multimodal pharmacotherapy. This case emphasizes the need to consider ACNES in patients endorsing focal abdominal pain with concurrent use of glucagon-like peptide-1 therapy and highlights injection site modification as a simple, effective management strategy while exercising self-restraint with respect to workup.

## INTRODUCTION

Anterior cutaneous nerve entrapment syndrome (ACNES) is an underdiagnosed cause of chronic abdominal pain, that represents a substantial proportion of abdominal wall pain referrals (not general population).^1–3^ ACNES is characterized by severe, often refractory, abdominal pain, due to entrapment of cutaneous branches of the lower thoracoabdominal (T8–T12) intercostal nerves, notably at the lateral border of the rectus abdominis (RA) muscle. These lower intercostal nerves are hypothesized to make an abrupt turn in their course through the RA channel, rendering them susceptible to entrapment leading to ACNES. In addition, muscle contraction at this location may cause additional nerve compression, resulting in severe pain due to mechanical or ischemic irritation.^3^ Entrapment can also happen from scar tissue, blood, suture, and exogenous liquid or solid.

In recent years, use of glucagon-like peptide-1 (GLP-1) receptor agonists for the management of diabetes mellitus and obesity has gained popularity. Although multiple causes have been hypothesized for the onset of ACNES, subcutaneous GLP-1 agonist injections triggering ACNES have not been documented in the literature. We report an unusual case of ACNES associated with subcutaneous administration of a GLP-1 agonist. A written informed consent was obtained from the patient for the publication of this case report.

## CASE REPORT

A 44-year-old man with obesity presented after being referred by his gastroenterologist for evaluation of abdominal pain. The onset of focal left-sided abdominal pain was noted 4 months prior, after initiation of an unspecified dose of a subcutaneous GLP-1 agonist (likely starting with 2.5 mg tirzepatide once weekly for 4 weeks) for obstructive sleep apnea. He had rotated the injection sites between the lower quadrants but stayed in RA territory. The sharp pain was localized to the sites and was distinct from his irritable bowel syndrome symptoms. He denied the history of nausea, vomiting, association of abdominal pain with bowel movements, surgical history, and abdominal trauma. During initial evaluation, he reported feeling depressed due to pain and was taking antidepressants for his mood. Furthermore, he was also trialing as needed tramadol for his neuropathic pain started 2 months after the onset by the referring gastroenterologist, in addition to another opioid for a preexisting oral palate lesion. His abdominal examination was notable for mild distension, increased tympany, extreme focal tenderness to palpation in the anterior cutaneous nerve distribution over his left abdomen, and a positive Carnett sign. No diagnostic imaging was obtained for the ACNES diagnosis to be ascertained.

He was recommended to continue tramadol along with lidocaine patches. He was also counseled to avoid the GLP-1 agonist injections in the abdomen and to instead use the lateral aspect of his arms. Upon his 4-month follow-up visit, he reported significant pain improvement with reduction in tramadol consumption. The tramadol was substituted with a voltage-gated sodium channel inhibitor which was now 6 months after the onset of his pain. He only received a month's supply at the last follow-up 9 months ago and has been estimated to be off the voltage-gated sodium channel inhibitor for about 8 months currently. At the last follow-up about 8 months after the pain onset, his pain had significantly improved (mild in intensity), but not fully resolved, suggesting possible permanent nerve entrapment.

## DISCUSSION

Chronic ACNES pain presents as a persistent dull ache ranging from mild to severe.^4^ Women are 4 times more predisposed to ACNES, with 2 distinct incidence peaks in the 15–20 and 35–45 year age ranges.^5^ Abdominal sensory innervation is regulated by the intercostal nerves, situated between the internal oblique and transversus abdominis muscles. The anterior cutaneous branches of the lower intercostal nerves (T8–12) traverse from 5 discrete points positioned medially to the linea semilunaris (where are patient initiated directed his injections) and enter the RA muscle posteriorly at a perpendicular angle. These nerve branches extend anteriorly and traverse a fibrous ring within the posterior sheath of the RA muscle. Considering these anterior branches enter the back of the muscle at nearly a right angle, they are more susceptible to mechanical irritation in addition to being increasingly vulnerable to external influences.^4^ Although pain is a predominant characteristic of ACNES, approximately 50% of patients present with other visceral manifestations such as bloating, nausea, and altered defecation.^6^ Symptoms of ACNES may be acute or chronic. The acute pain is described as localized, dull, or burning, with a sharp component (usually unilateral) and pain radiation with movements such as twisting, bending, sitting up, or, in some cases, even with lying down. The patient described in this study, had the classic presentation of ACNES with pain in the distribution of an ACN-innervated region with focal tenderness to palpation. Usually, while examining a patient with abdominal pain, diagnostic tests are often used to evaluate for abdominal visceral disorders. However, in a patient with ACNES, these tests are usually normal, which makes it more likely they will be misdiagnosed as functional abdominal diseases or psychiatric disorders. The management of ACNES varies from pharmacotherapeutic management (gabapentin, amitriptyline, etc.) to peripheral nerve stimulation, spinal cord stimulation, or dorsal root ganglion stimulation to invasive surgical procedures such as partial rhizotomy and anterior neurectomy.^7^

Subcutaneous injections involve injection into subcutaneous fat. These injections are usually self-administered and can be administered with minimal training. GLP-1 receptor agonists are an effective treatment option for patients with obesity and type 2 diabetes as they stimulate insulin secretion while suppressing glucagon secretion in hyperglycemic states while also delaying gastric emptying, thereby reducing appetite and body weight.^8^ They are available as oral tablets or subcutaneous injections. Tirzepatide is a dual glucose-dependent insulinotropic polypeptide and GLP-1 receptor agonist available as a subcutaneous injection.^8^ Clinical evidence from trials and real-world scenarios report a high prevalence of gastrointestinal side effects with the use of GLP-1 agonists, with the most common being nausea, vomiting, gastroparesis, diarrhea, constipation, and pancreatitis (less likely)^9^; but in this case, administration of subcutaneous GLP-1 agonist injections coincided with the onset of ACNES, possibly by irritation of the anterior cutaneous branches. A PubMed literature search for any previous publication detailing the onset of ACNES with the use of GLP-1 agonist injections was negative, but there are 2 cases of related neuropathic pain 10. To the best of our knowledge, this is the first case report describing the onset of ACNES following administration of GLP-1 agonist injections.

A previously reported case involved 2 subcutaneous GLP-1 agonists that resulted in dysesthesia at unspecified location several weeks after transitioning to the second agonist with spontaneous resolution within 6 weeks. The second case involved a patient with diabetes mellitus type 2 and neuropathy who developed diffuse allodynia after transition from 1 GLP-1 agonist to tirzepatide. There was spontaneous resolution within 2 weeks, with 2 recurrences that resolved with transition of the GLP-1 agonist to an oral one. Regardless, oral semaglutide has been associated with dysesthesia, hyperesthesia, paresthesia, burning sensation, and neuralgia in 22 clinical trials.^10^ Moreover, even before Ahern cases, GLP-1 agonists had been associated with allodynia and dysesthesia.^11,12^ The effect may be dose-dependent, occurring at the highest doses. In some cases, spontaneous resolution occurred 6–16 weeks after onset. In the aforementioned 2 patients, symptoms resolved when the GLP-1 agonist was stopped, while 1 patient experienced after it was resumed with resolution after 4 months.^10^

Albeit the temporal relationship between abdominal injections and symptom onset suggests a potential association, causality cannot be definitively determined. It is plausible that repetitive use of the same injection sites resulted in localized nerve irritation commonly seen in subcutaneous administration vs true mechanical nerve entrapment. Nevertheless, symptom improvement following injection site modification supports a clinically meaningful association.

It is important to note that subcutaneous injections play a prominent role in the lives of patients with gastrointestinal disorders, especially those with inflammatory bowel disease who have to self-administer biologics. It is important to educate patients to look for sudden onset pain with neuropathic qualities: sensation of burning, sunburn, stinging, pins and needles, electricity, tingling, and even pruritus. The injection site should be changed immediately, and the patient should be reminded of sites that do not contain nerves such as the anterior cutaneous nerves or the axillary nerves in the lateral arms.

We conclude by emphasizing that ACNES should be considered as a potential differential for abdominal pain associated with GLP-1 injections, with equal emphasis on changing the site of subcutaneous injections and exercising self-restraint with respect to the diagnostic workup.

## DISCLOSURES

Author contributions: N. Singh: Writing–Original Draft; Review. E. Chaparro: Writing–Original Draft, Review and Editing. EN Husu: Conceptualization; Supervision; Review and Editing. JM Shapiro: Conceptualization; Supervision; Review and is the article guarantor.

Financial disclosure: None to report.

Informed consent was obtained for this case report.

## ABBREVIATIONS:

ACN: anterior cutaneous nerve
ACNES: ACN entrapment syndrome
GLP-1: glucagon-like peptide-1
RA: rectus abdominis
